# RFID Technology for Management and Tracking: e-Health Applications

**DOI:** 10.3390/s18082663

**Published:** 2018-08-13

**Authors:** Yuri Álvarez López, Jacqueline Franssen, Guillermo Álvarez Narciandi, Janet Pagnozzi, Ignacio González-Pinto Arrillaga, Fernando Las-Heras Andrés

**Affiliations:** 1 Área de Teoría de la Señal y Comunicaciones, Universidad de Oviedo, 33203 Gijón (Asturias), Spain; alvareznguillermo@uniovi.es (G.Á.N.); flasheras@uniovi.es (F.L.-H.A.); 2 Faculty of Computer Science, Hochschule Mannheim, 68163 Mannheim, Germany; jacqueline.franssen@stud.hs-mannheim.de; 3 Departamento de Medicina, Universidad de Oviedo, 33006 Oviedo (Asturias), Spain; UO253695@uniovi.es (J.P.); igpinto@uniovi.es (I.G.-P.A.)

**Keywords:** radio frequency identification (RFID), e-health, radioelectric coverage, internet-of-things, indoor location systems (ILS), assets tracking, logistics

## Abstract

Radio frequency identification (RFID) has become a key technology in the logistics and management industry, thanks to distinctive features such as the low cost of RFID tags, and the easiness of the RFID tags’ deployment and integration within the items to be tracked. In consequence, RFID plays a fundamental role in the so-called digital factory or 4.0 Industry, aiming to increase the level of automatization of industrial processes. In addition, RFID has also been found to be of great help in improving the tracking of patients, medicines, and medical assets in hospitals, where the digitalization of these operations improves their efficiency and safety. This contribution reviews the state-of-the-art of RFID for e-Health applications, describing the contributions to improve medical services and discussing the limitations. In particular, it has been found that a lot of effort has been put into software development, but in most of the cases a detailed study of the physical layer (that is, the characterization of the RFID signals within the area where the system is deployed) is not properly conducted. This contribution describes a basic RFID system for tracking and managing assets in hospitals, aiming to provide additional details about implementation aspects that must be considered to ensure proper functionality of the system. Although the scope of the RFID system described in this contribution is restricted to a small area of the hospital, the architecture is fully scalable to cover the needs of the different medical services in the hospital. Ultra high-frequency (UHF) RFID technology is selected over the most extended near-field communication (NFC) and high-frequency (HF) RFID technology to minimize hardware infrastructure. In particular, UHF RFID also makes the coverage/reading area conformation easier by using different kinds of antennas. Information is stored in a database, which is accessed from end-user mobile devices (tablets, smartphones) where the position and status of the assets to be tracked are displayed.

## 1. Introduction

The rapid development of information communication and technology (ICT) has made possible the automatization of human-assisted tasks in a wide range of applications, such as asset tracking for logistics (mainly within the context of 4.0 Industry) or people monitoring for security and safety applications. In recent years, the Internet of Things (IoT) [1,2] and sensor networks [2,3,4] have contributed dramatically towards this goal.

One of the reasons for increasing the automation level in low- and medium-complexity tasks is to reduce the risk of mistakes made by employees. Routinely tasks are under supervised due to an overestimation of the level of self-confidence of the employees. In sectors such as health, this is especially critical, as it has a direct impact in the health condition of the patients. For example, medication errors are behind 22,300 deaths per year in the National Health Service (NHS) [5], having also a significant economic impact [6].

Several factors related to medication error can be highlighted [7]: (i) similarity in the packaging or names of the drugs; (ii) patients allergic to some drugs; (iii) drugs whose use is very specific. Up until 2008, the wrong route of drug administration, wrong medication, and wrong dose were responsible for 9.5%, 16%, and 40.9% of patient death due to medication errors, respectively [7]. These figures support the need for automation and/or the introduction of additional ICT supervision systems in the aforementioned medical procedures.

The implementation of ICT systems for automation of logistics and management in hospitals should consider the following design requirements [2,8,9]:

(i) the use of disposable, low-cost sensors, which will be tagged to the patients, medication, and medical assets to be tracked; and

(ii) the connectivity between hardware (readers, end-user mobile devices, servers with their databases located at the hospital ICT department) should be adapted to the existing network infrastructure of the hospital and/or be based on wireless networks.

Among the advantages provided by ICT systems for location and tracking in healthcare applications [2], the following can be noted:

(i) Minimization of medical errors: on the one hand, patients labelled with a RFID wrist can be tracked in real-time, so doctors, physicians and nurses can know if there is an issue (e.g., a delay) in the medical treatment required by the patient. On the other hand, drugs can be better tracked, thus reducing the risk of wrong or forgotten drug administering to patients.

(ii) Optimization of resources: for example, doctors and nurses can know the exact amount of blood supplies. Some medical assets and instrumentation can also be located easily, including information about their availability.

ICT systems can be grouped according to the most extended technologies: optical identification systems (barcodes and QR codes) [10] and radiofrequency-based systems [11].

The use of QR codes and barcodes for traceability and logistics has been introduced in a wide range of sectors for decades, and also in the field of medicine (e.g., traceability of patients during the different stages of the medical treatment at the hospital). Aiming to overcome some of the limitations of these optical-based tracking systems (listed in Table 1), radiofrequency-based technologies have been developed and gradually implemented, first in the area of transport and logistics (e.g., toll collection in highways, warehouses and retail stores), and recently in the field of medical services (e-health).

Radiofrequency-based systems for location and tracking consist mainly of tags (attached to the items or patients to be monitored) and tag readers. Two technologies can be distinguished: on the one hand, high-frequency (HF) radio frequency identification (RFID) [12] and near-field communication (NFC) [12] systems, in which wireless information transfer between the tags and the reader is based on electromagnetic field coupling [13,14]; and on the other hand, ultra high-frequency (UHF) RFID systems, in which electromagnetic backscattering is used to send information from the RFID tags to the reader [13,14]. Table 1 summarizes the advantages and disadvantages of NFC/HF RFID and UHF RFID technologies, also compared to optical systems.

Several examples of RFID systems already deployed in hospitals and medical services can be found in the literature [15,16], some of them highlighted in Table 2. For example, [15] presents a medication administration system which automatically verifies medication and generates the corresponding prescription, aiming to avoid errors related to paper-based procedures. After deploying the application, it was reported that the number of medical mistakes was significantly reduced. The introduction of a RFID system is described by [17] for a better control of medical assets, providing faster response time (as the doctors and nurses spend less time searching for medical devices). Another example of fully operational RFID system for tracking and managing drugs and patients is presented in [18]. In this case, a dual NFC/UHF RFID system is proposed to ensure the detectability and also to make traceability easier. In addition to this, the level of automation with respect to handheld NFC readers is improved.

A hybrid infrared/RFID real-time location system (RTLS) is described in [20], providing detection ranges up to 2 m even in non line-of-sight (NLOS) conditions. This system was initially conceived for monitoring and tracking medical items (e.g., to detect those requiring maintenance), and later extended to track nurses when they respond to emergency calls or visit patients.

In most of the RFID-based systems for healthcare applications presented in Table 2, a survey has been conducted to evaluate the level of satisfaction and comfort of the employees (doctors, nurses) and patients. In general, there is a good acceptance of these systems as they effectively contribute to reduce medical errors, enhancing patients’ monitoring and attention during hospitalization. The main complaints come from the need of adoption of new medical protocols (training of doctors and nurses), especially if they require additional tasks (e.g., approaching the items tagged to the NFC or RFID reader). This problem could be mitigated if UHF RFID systems, with longer reading range, were used.

The compatibility and coexistence of the RFID-based system with already implanted ICT systems in the hospital is also an important issue [2,22]. To achieve this goal, it is suggested to start with small RFID projects [15] (as the proof-of-concept presented in this contribution) ensuring that the tested system can be later scaled up to cover the entire hospital needs.

Another issue, missed in the deployment and evaluation of the RFID systems cited in the literature review, is a proper characterization of the physical layer, such as the coverage of the RFID antennas or the selection of the RFID antennas’ placement, to fulfill coverage and detectability requirements.

### Aim and Scope

This contribution aims to provide detailed information concerning the implementation and testing of a basic RFID system for e-Health applications, in particular, for tracking and managing assets (medication and patients) in hospitals. While the architecture of the proposed system is quite similar to those already deployed in hospitals worldwide, the aim here is to provide additional details about implementation features that must be taken into account to ensure proper functionality: (i) choice of the antennas depending on the coverage area and cost; (ii) item traceability depending on the RFID tags’ detectability; and (iii) simplicity and ease of use of the graphical user interface (GUI). Although the scope of the RFID system described in this contribution is restricted to a small area of the hospital, the architecture is fully scalable to cover the needs of the different medical services in a hospital.

The implemented system allows the tracking of medical assets that are tagged with RFID tags within a hospital. UHF RFID technology is selected over the most extended NFC and HF RFID technology to minimize the hardware infrastructure. The former also allows ease of coverage/reading area conformation by simply using different kind of antennas. Information is stored in a database, which can be accessed from end-user mobile devices (tablets, smartphones) where the position and status of the assets to be tracked is displayed.

## 2. System Architecture

### 2.1. Radio Frequency Identification (RFID) Hardware and Considerations about Signal Propagation

As outlined in Table 2, the main advantage of UHF RFID over HF RFID systems is the capability of providing longer reading distances, thus minimizing the need for approaching items to the readers. Conventional UHF RFID antennas and readers can provide a reading distance up to 6–7 m far from the antenna even in NLOS and multipath conditions, so a single UHF RFID antenna is enough to provide coverage within a hospital room.

UHF RFID-based systems for management and tracking applications consist of [12,13]: (i) a set of UHF antennas; (ii) one or several RFID readers; (iii) a computer that manages the RFID readers; and (iv) UHF RFID tags attached to the items to be tracked. While the cost of a RFID tag is less than 0.05 EUR, UHF antennas are within the range of 20–200 EUR (depending on their specifications and requirements), and UHF RFID readers are in the range of 200–1100 EUR also depending on their specifications and performance. The use of antenna hubs enables increasing the number of antennas that can be controlled by a single RFID reader, thus cutting down the cost of the overall system. Typical specifications/performances of a reader involve the number of tags read per second, or the number of UHF antennas that can be controlled.

**Figure 1:**
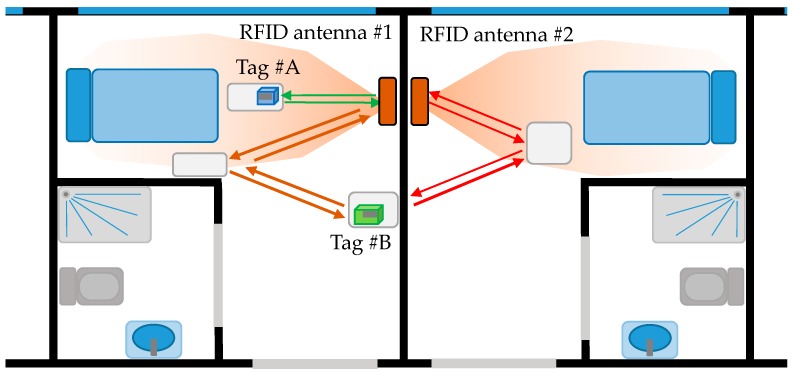
Example of non-desired RFID tag read due to multipath (*in-channel* interference). Item tagged with tag #A is within the coverage area of RFID antenna #1, while item tagged with tag #B is located outside the coverage area. Green arrows represent desired interrogation/response. Brown arrows show non-desired interrogation/response due to multipath affecting RFID antenna #1. Red arrows represent non-desired interrogation/response due to multipath affecting RFID antenna #2 (in this case, interrogation and response RFID signals are partially attenuated when passing through the wall).

RFID antennas are placed to provide a coverage area within a place of interest, for example around the beds in hospitalization rooms (4–5 m^2^). It is important to avoid coverage areas overlapping to ensure that RFID tags are detected by just one antenna. Another source of error is the presence of multipath [23,24], especially in indoor scenarios like the one illustrated in Figure 1. Under normal operation conditions, only those items within the coverage area of the RFID antenna are detected. However, multipath can result in the detection of RFID tags beyond the coverage area of the antenna (*in-channel* interference [25]). To overcome this problem, the following solutions can be adopted: (i) reducing the power transmitted by the RFID antenna while ensuring that the minimum level to detect RFID tags is achieved within the coverage area; (ii) using antennas whose radiation pattern fit the area of interest, thus minimizing reflections. For example, directive antennas are considered in the example shown in Figure 1 to provide coverage around the beds. The use of directive antennas also contributes to mitigate multipath contributions.

**Figure 2:**
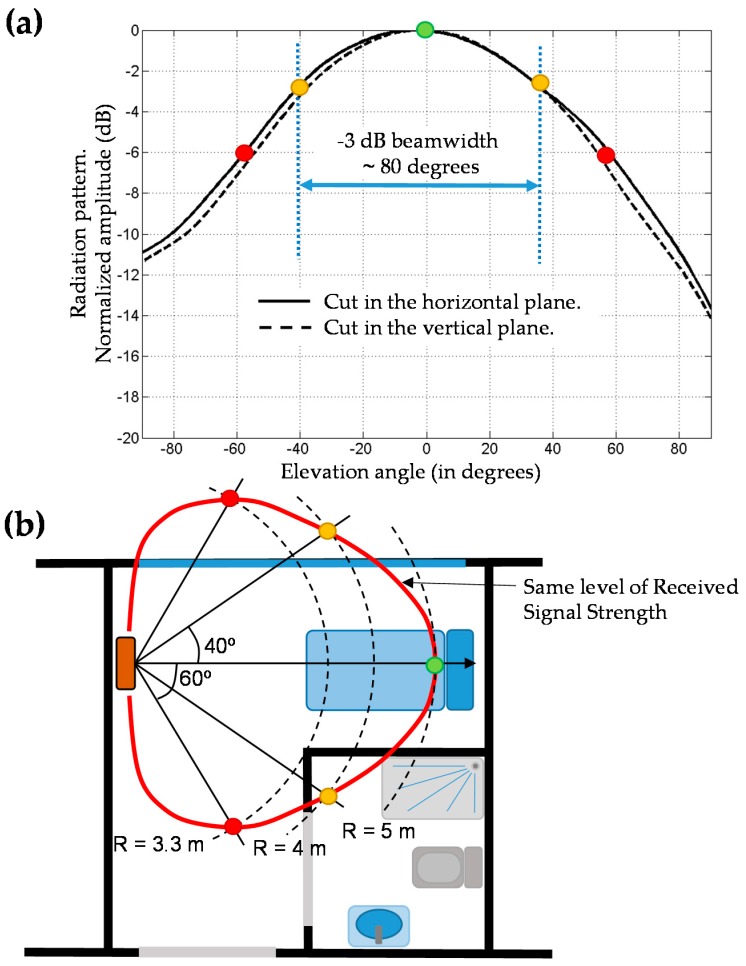
(**a**) Radiation pattern of a directive UHF RFID. Main cuts are depicted, highlighting the −3 dB beamwidth. (**b**) Representation of the coverage area of the RFID antenna: red line represents all the points that receive the same signal strength taking into account indoor propagation model losses and the radiation pattern.

To illustrate the influence of the antenna radiation pattern, Figure 2a shows the main cuts of the radiation pattern of a RFID antenna [26], that has around 80° −3 dB beamwidth in both planes (resulting in ~8.1 dBi directivity). Given the radiation pattern, it is possible to obtain an estimate of the coverage area considering propagation losses (*Att*). These losses are estimated according to a log-distance path loss model. Among the different methodologies and models proposed for indoor channel characterization [27,28], the International Telecommunication Union Radiocommunication Sector (ITU-R) path loss definition [29] has been selected as a trade-off between accuracy and complexity (1):

(1) $$Att\left(R\right)\left(\mathrm{dB}\right)=20\;log_{10}\left(f\right)+nlog_{10}\left(R\right)+Lf-28\;\mathrm{dB}$$

where *f* is the working frequency (in MHz) of the UHF RFID system (868 MHz in Europe), *R* is the distance from the transmitter to the tag, *n* is the path loss exponent (20 for free-space propagation, 33 for an indoor environment such as the one described in this contribution), and *Lf* accounts for signal attenuation when passing from one floor to another (*Lf* = 0 dB in this case, as the RFID reader and RFID tags are on the same floor).

An example is presented in Figure 2b. Let us suppose that the UHF RFID antenna reading range reaches *R* = 5 m at its pattern maximum (green dot in Figure 2b). At an angle of 40°, the radiation pattern decays 3 dB. Thus, compensable attenuation for this pattern level is given by (2):

(2) $$Comp.Att\left(\mathrm{dB}\right)=Att\left(R_{1}\right)-Att\left(R_{2}\right)$$

with $R_{1}$ = 5 m and *Comp.Att* (dB) = 3 dB. Applying Equation (1), $Att\left(R_{1}=5\;m\right)$ = 53.8 dB, so that $Att\left(R_{2}\right)$ = 50.8 dB, yielding $R_{2}$ ~4 m.

Equation (2) can be used to predict the angle at which the same signal level as at the pattern maximum is reached given the distance from the antenna. For example, for $R_{2}\;$= 3.3 m, $Att\left(R_{2}\right)$ = 47.8 dB, yielding *Comp.Att* (dB) = 6 dB. From Figure 2a, this corresponds to an angle of approximately 60°. Repeating this procedure for different angles and/or distances, an estimate of the UHF RFID antenna coverage area in the scenario to be deployed can be obtained, allowing users to decide whether this kind of antenna fulfills the coverage requirements or not.

In addition to this, the power level of the RFID reader can be adjusted in most of commercial UHF RFID readers, so it can be tuned to modify the maximum reading distance (e.g., 5 m in the case of Figure 2b example).

**Figure 3:**
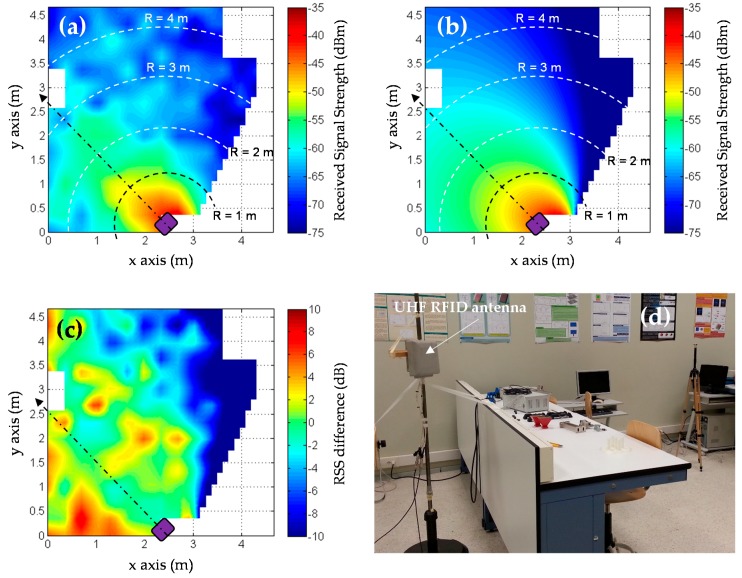
Experimental evaluation of UHF RFID antenna coverage, that was conducted for the research presented in [30]. (**a**) Measured received signal strength (RSS); (**b**) Estimated RSS considering an indoor propagation losses model and the UHF RFID antenna pattern; (**c**) Difference between measured and estimated RSS; (**d**) Picture of the scenario where antenna coverage was assessed.

A better way to assess RFID coverage in the scenario where the system is going to be deployed is, of course, by means of measurements. In the case of RFID antennas, coverage can be evaluated empirically by simply placing one or several RFID tags at those positions of interest, recording the values of received signal strength (RSS) and then plotting the RSS values over a plane of the scenario [30]. An application example is shown in Figure 3, where RSS measurements, conducted in the scenario shown in Figure 3d, were compared to theoretical RSS values calculated considering an indoor propagation losses model [30] and taking into account the radiation pattern of the UHF RFID antenna [30]. The effect of multipath can be clearly observed in the measurements (Figure 3a): signal strength does not monotonically decay with distance. Of course, more accurate methodologies for coverage evaluation (such as the proposed in [24]) can be considered depending on the system requirements.

In general, RFID tags exhibit a dipole-like radiation pattern [31] with linear polarization. Because of this, most UHF RFID antennas are circularly polarized, so that the received signal strength is quite independent from RFID tag orientation with respect to the UHF RFID antenna.

Another issue is the performance of RFID tags depending on the surface where they are placed. Low-cost UHF RFID tags do not perform well when placed on metallic containers or boxes, or recipients containing liquids, thus requiring a special kind of RFID tag (whose cost is typically 2–50 times higher). In the case of hospitals, most of the drugs are presented in cardboard or plastic containers, allowing the use of low-cost, conventional RFID tags.

Coaxial cables are used to connect UHF antennas to the readers. In order to reuse existing hospital infrastructure, and thus minimize the cost of the RFID system deployment, connection between antennas and the RFID reader could be done using the radio and television distribution network based on 75 ohm coaxial cable. In that case, RFID antennas and readers which characteristic impedance of 75 ohm (instead of the most common 50 ohm) should be used. In Europe, the upper frequency band of radio and television broadcasting is more than 100 MHz below UHF RFID frequency band (865–868 MHz), so there would not be interferences between RFID and the signals of radios and televisions.

Next, to connect the RFID readers and the computer that manages them, the easiest solution is to provide local area network (LAN) connectivity, since most of the hospitals have several public and protected LAN domains, depending on the targeted users (visitors and patients, doctors and physicians, etc.). It must be pointed out that most of current RFID readers existing in the market can be connected via Wi-Fi (e.g., by means of an Ethernet/Wi-Fi Access Point), thus reducing the need for deploying additional Ethernet cable infrastructure.

### 2.2. RFID Software

RFID software architecture can be divided into two parts: the first one is devoted to managing the readings of RFID tags within the coverage area of the antennas, storing and updating the information in a database. The second one includes the GUI where the information stored in the database is displayed to end-users and administrators, as well as allowing the collecting of their inputs.

**Figure 4:**
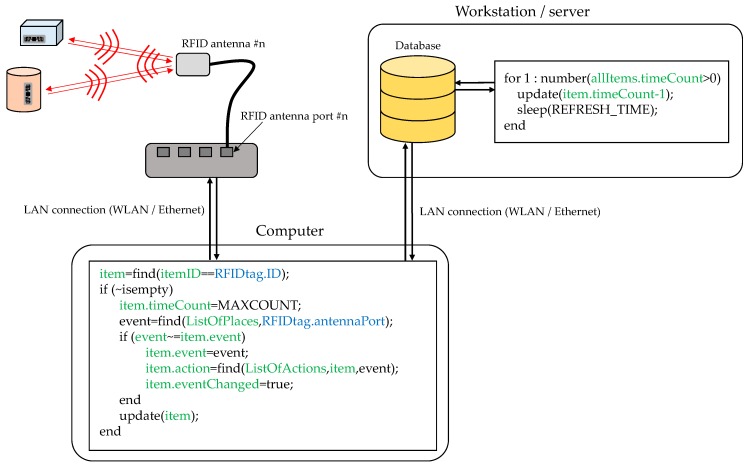
Scheme of the connection between the RFID antenna, the RFID reader, the computer controlling the RFID reader, and the system database. Pseudo-code illustrating how the database is updated every time a RFID tag is read is outlined (information associated to the database is highlighted in green, while inputs from RFID reader are highlighted in blue).

**Figure 5:**
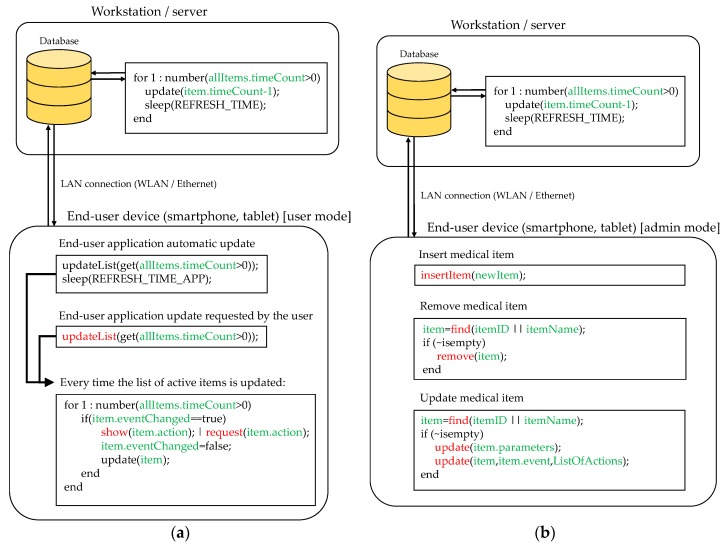
(**a**) Pseudo-code showing the interaction between an end-user and the information stored in the database. (**b**) Pseudo-code illustrating how new items are introduced in the database when the user is authenticated as administrator. Information associated to the database is highlighted in green, while end-user actions using the mobile application are denoted in red.

RFID tags (especially passive ones) can store a limited amount of information about the associated item. Because of this, RFID tags are typically used as pointers to registers in a database where information about the item is stored. In the example shown in Figure 4 and Figure 5, each tagged item has an associated entry in a database containing the following information: (i) *item ID:* RFID tag identifier or pointer; (ii) *item name:* name of the medical asset or associated medicine/drug; (iii) event: indicates where the RFID tags are detected; (iv) *action:* depending on the kind of item and the event, different actions are triggered (e.g., administering the item to the patient on the room); (v) *eventChanged:* flag that tells if, during the period of two consecutive readings, the event has changed (e.g., if the item has been moved from one room to another); (vi) *timeCount:* counter to consider if the item is active or not within the coverage area of an antenna. This database can be stored in a workstation/server, eventually integrating them together with other information systems and services of the hospital.

RFID readers are configured to periodically send interrogation signals, each one associated to the n-th RFID reader port (which is connected to the n-th RFID antenna). If a RFID tag receives this interrogation signal with enough intensity, it will send a response signal. Collision avoidance protocols are implemented for those cases in which two or more RFID tags respond at the same time to the interrogation signal. That means that only one response is received every time. This could result in missed tags for those cases where there are tens of RFID tags within the coverage area of the antenna and the RFID reader throughput is low. This also leads to another problem associated with latency.

For example, if there are 10 RFID tags within the coverage area of the n-th antenna, and the RFID reader throughput is 10 tags per second, each tag is detected 1 time per second on average. Thus, a protocol that tells the end-user that the tag is within the coverage area, although it is not constantly read, must be implemented. The solution proposed in this contribution is shown in Figure 4: every time a RFID tag is read, the field *timeCount* is set to its maximum value (*MAXCOUNT*). Then, the value of this counter is decreased periodically in the database. If the value of the counter reaches zero, then it is considered that the item is no longer within the coverage area of the antenna. However, if a new reading is done before the counter reaches zero it is set back to *MAXCOUNT*. The value of *MAXCOUNT* can be adjusted depending on the throughput of the RFID readers and the expected maximum number of RFID tags within the coverage area. Thanks to this, items can be considered “active” for a few seconds in the location associated to the n-th antenna where they have been detected.

RFID tags attached to items that are not stored in the database (e.g., clothing of hospital visitors) will be detected as well, thus reducing the effective throughput of the readers. This issue must be considered when selecting the value of *MAXCOUNT*. As shown in Figure 4, once a RFID tag is detected, the first action is to check whether the item associated to the tag exists in the database and, otherwise, the reading is discarded.

The information stored in the database eventually has to be displayed to end-users (doctors, physicians) and system administrators. Hospitals including e-health procedures usually provide mobile devices (such as smartphones, tablets) to their employees, giving them access to real-time information about patients, supplies, etc. In this regard, mobile applications for these devices have been implemented to make easier the visualization of the information and the collecting of inputs. Furthermore, these devices can be connected to the hospital wireless LAN to access different information systems (such as the RFID system database), thus avoiding the need for additional network infrastructure.

Figure 5 illustrates two basic functionalities of the proposed mobile application. Figure 5a corresponds to user mode functionality. In this configuration, the mobile application shows those items considered “active” within one of the coverage areas of the RFID system (that is, *timeCount* > 0). The list of active items is periodically refreshed (controlled by the variable *REFRESH_TIME_APP*). Besides, the user can manually request an update of this list (Figure 5a, function *updateList*). Every time the list is updated, the application checks if there was an update in the event associated to each active item and, if so, the application checks if there is an action required, whose type will depend on the event and the kind of detected item.

To illustrate this, let us suppose that a box containing ibuprofen has to be administered to the patient in room 312. First, this item is detected in the hallway (event: “detected on hallway”). Later, a nurse goes into room 312 carrying the box of ibuprofen, so when the box is within the coverage area of the RFID antenna located in room 312, the event in the system database is updated to “detected in room 312”. Then, taking into account the event (“detected in room 312”) and the item (“ibuprofen”), the system checks if there is an action that should be performed (for example, “administer medicine to patient”). If so, the system will require the end-user to complete the action, e.g., by displaying a message in the mobile application (together with another kind of alarm such as smartphone vibration or short ring). Once the end-user completes the required task, the action associated to the item is updated.

Moreover, the mobile application can be used for administration purposes. In this case, the user must log in as administrator. Three basic actions are considered (see Figure 5b): (i) register a new medical item in the database; (ii) remove an existing medical item; and (iii) update the information associated to the item (e.g., action to be done when a certain event happens). Of course, more complex functionalities can be added, but these are beyond the scope of the proof-of-concept presented in this contribution.

## 3. Application Example

### Description of the Scenario

**Figure 6:**
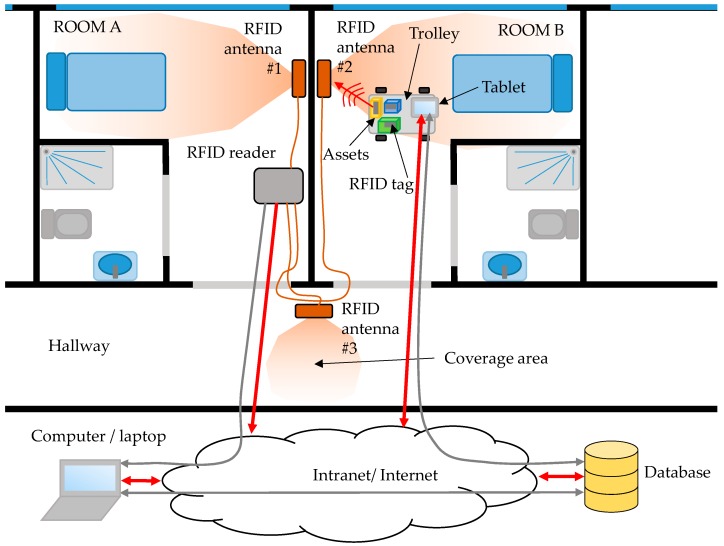
Scheme of the RFID system architecture used for the proof-of-concept.

**Figure 7:**
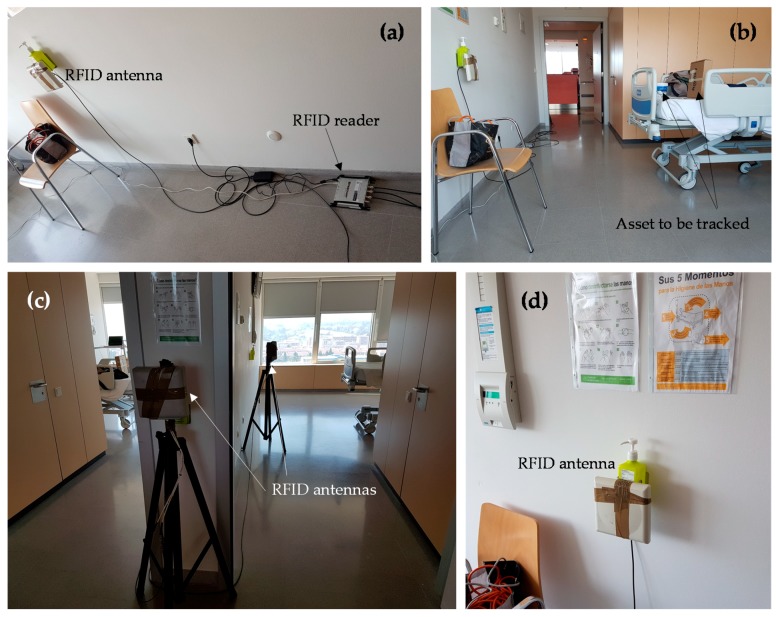
Pictures of the scenario where the RFID system has been deployed (rooms 312, 314, and hallway of the “Hospital Universitario Central de Asturias”). (**a**,**b**) Room 312. (**c**) Hallway and room 314. (**d**) Close-up of the placement of a RFID antenna in room 312.

In order to illustrate how the RFID system proposed in this contribution works, a simple demonstration has been conducted in the Central Hospital of Asturias (“Hospital Universitario Central de Asturias”, HUCA, Asturias, Spain). The RFID system has been deployed in rooms 312 and 314, as well as in part of the hallway of the third floor, as depicted in Figure 6. One RFID antenna has been placed on each room, providing a 3 m × 5 m coverage in front of the antenna, which corresponds to the area of the room where the bed and the medical instrumentation are placed. Although RFID antenna models are different (see Figure 7), they have a radiation pattern very similar to the one shown in Figure 2a, which is actually the radiation pattern of the antenna depicted in Figure 3d [26], measured at the spherical range in anechoic chamber of the University of Oviedo. The coverage area has been estimated based on the methodology described in Section 2.1. Besides, the power level of the RFID reader has been adjusted to reduce the maximum reading distance up to 5 m, providing RFID tags’ detection capabilities even in NLOS within the coverage area, and ensuring that RFID tags outside the reading zone will not respond to RFID reader interrogations.

Three RFID antennas have been connected to an Impinj Speedway Revolution 4-port RFID reader [32] that provides at least 50 readings per second (depending on the reader configuration parameters, number of antennas connected to it, etc.). The RFID reader is connected to a laptop that manages it, that is, that sets up the reader and collects the events triggered every time a RFID tag is detected, updating the database accordingly. The locations of the RFID antennas and the RFID reader are depicted in Figure 6 and Figure 7.

**Figure 8:**
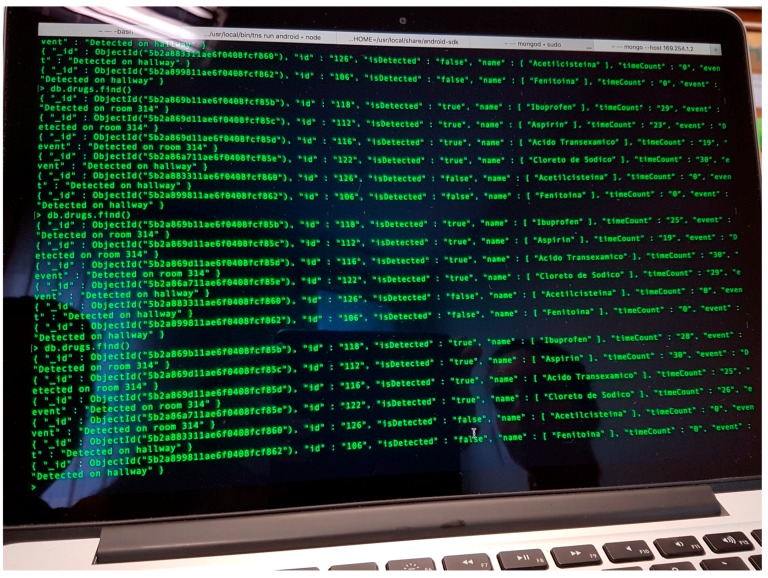
Screenshot of the MongoDB software [33], showing a list of the items registered in the database, together with their attributes.

The database has been installed on a second laptop, aiming to have a system architecture resembling a real system, where the database would be set up in a workstation on the computer service area of the hospital. In this proof-of-concept, the database has been implemented in MongoDB (Figure 8) [33], as it is one of the most widespread SQL database softwares nowadays. An Ethernet LAN has been set up to connect the RFID reader and the two laptops.

**Figure 9:**
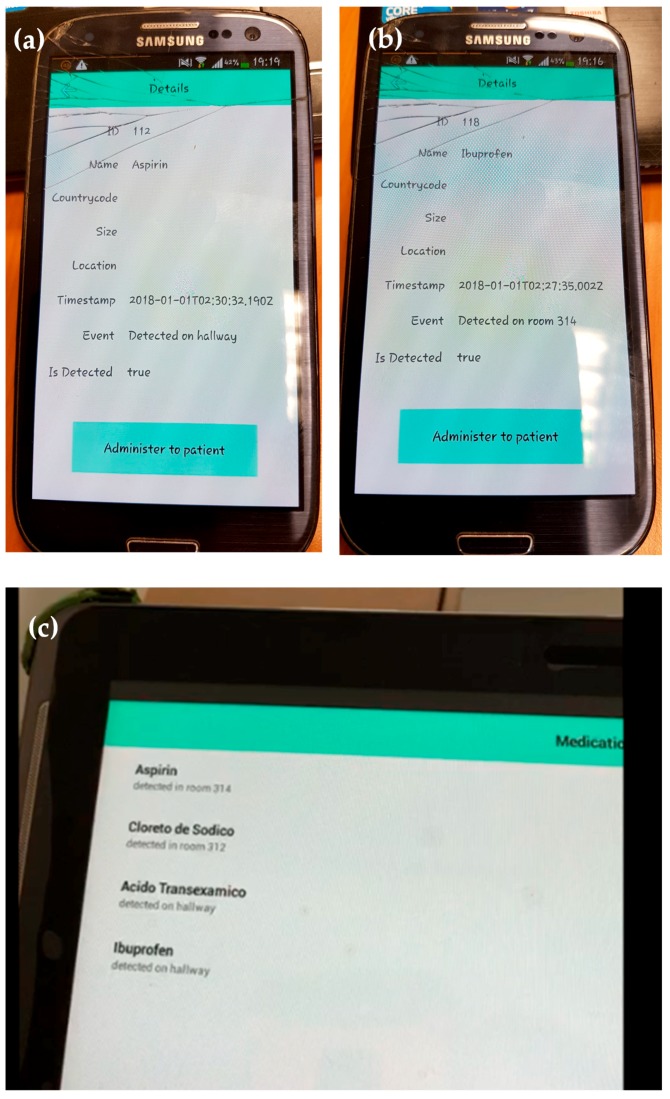
Screenshots of the mobile application implemented to show real-time information of the tagged assets detected by the RFID system. (**a**,**b**) Mobile application installed in a smartphone, showing detailed information about the item detected. (**c**) Mobile application installed in a tablet, showing the list of detected items and where they are.

The end-user mobile application has been installed on two smartphones (Samsung Galaxy S3 with Android 4.3, iPhone 7 with iOS 11.4) and in a tablet (Samsung GT-P5110 with Android 4.2.2). This application has been developed in NativeScript [34] to provide support to mobile devices based on both iOS and Android platforms. The application developed for this proof-of-concept is very simple, consisting of a main window showing the list of items detected by the RFID antennas and the location (event) where they were detected (Figure 9c). If the user presses the name of an item, a second window is opened. This window provides detailed information (if available) about the tagged item, such as the ID of the RFID tag, medical information, debug information for the software developer (to be removed in a final version of the application), and the action to be done by the end-user (e.g., “administer medicine to patient”). Examples of this window with detailed information are shown in Figure 9a,b.

For the sake of simplicity, the mobile devices have been connected to the laptop hosting the database using a WLAN created ad hoc for the tests. However, the WLAN network available at the hospital could have been used to provide wireless interconnectivity.

**Figure 10:**
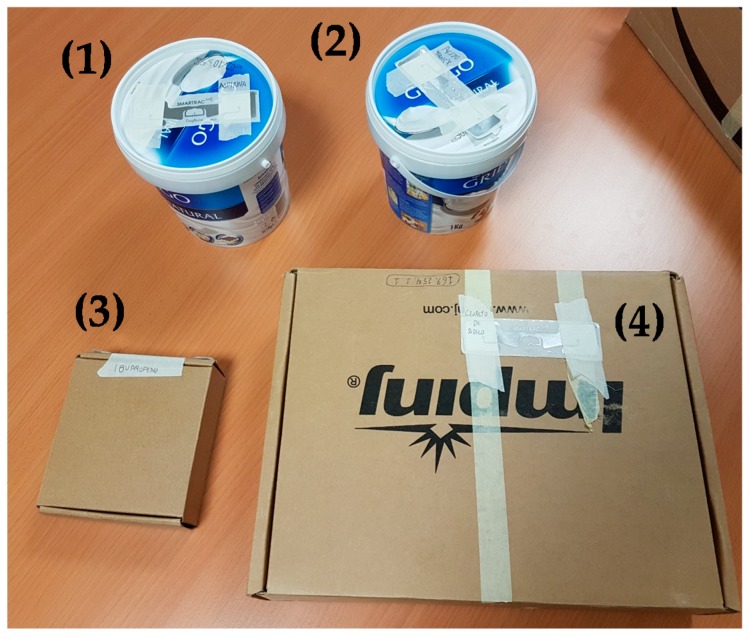
Examples of items tagged with passive DogBone RFID tags [31], emulating medical assets and drugs containers: (1) aspirin, (2) tranexamic acid, (3) ibuprofen, (4) sodium chlorate.

Five items (four are shown in Figure 10) were tagged with UHF RFID DogBone tags [31] in order to test the RFID system. These items were distributed between rooms 312 and 314, placing two of them on a cart used by nurses and doctors to transport medicines and medical items. The maximum number of RFID tags in these tests was not greater than 10, and the system was capable of processing 5 reads per second (taking into account the time required by all the elements of the system: RFID reader, database, and laptop acting as an interface between the database and the RFID reader). Thus, the variable *MAXCOUNT* was set to 30, decreasing the value of the field *timeCount* every 10 ms (*REFRESH_TIME* = 10 ms). That means that every time a RFID tag is read it is considered active for at least 3 s (if it is read before this timeout, then the field *timeCount* will be set back to 30, starting a new period of 3 s).

First, the capability of the system to detect the tagged items was tested. For this purpose, one item was left within the coverage area of the RFID antenna located in room 312, another one was left in room 314 within the coverage area of the antenna placed in that room, and the remaining items were left outside the coverage areas. As expected, only those two aforementioned items were detected, being shown in the mobile application installed on the tablet and in the smartphone.

Next, an item in room 312 was moved out of the coverage area. As expected, after 3 s since the last read, the field *timeCount* associated to this item reached a value of 0, and in consequence, the item was removed from the list of active items. Once the item was placed again within the coverage area it was shown in the list of active items, with the same associated event “detected in room 312”.

In the third test, two items were mounted on the cart, which was placed within the coverage area of room 312. As a result, the mobile application showed 4 detected items, 3 of them in room 312. Then, the cart was moved from room 312 to room 314 at a slow pace. As soon as the cart was within the coverage area of the antenna placed in the hallway, the event associated to the items in the cart was updated to “detected on hallway”, as shown in Figure 9c. When the cart was within the range of the antenna placed in room 314, the event associated to these two items was again updated, as shown in the following video (which also includes an overview of the system presented in this contribution): https://youtu.be/cvR7DlN-iUg (Supplementary Materials).

Additional tests were conducted proving the capability of the system to detect the tagged items and to update the events accordingly. No undesired readings due to the presence of multipath were reported during these tests.

## 4. Discussion

The RFID system presented in Section 2, although quite simple, shares several characteristics with others already operational in hospitals worldwide cited in the literature review of Section 1. As in those systems, the hardware front-end consists of several antennas placed in the areas of interest within the hospital connected to an RFID reader. Items and patients to be monitored are tagged with passive RFID tags. Information about the tags detected by the reader is sent to a database that stores the status of these tags and the associated items. Using a mobile application, end-users can retrieve information about the items in real-time (events, location, etc.).

The simple proof-of-concept shown in this contribution proves the feasibility and ease of implementation and use of these systems. In this case, it should be remarked that once the items are tagged with RFID tags, potential users (doctors, nurses) do not need to interact with the hardware front-end. Furthermore, RFID antennas and readers could be hidden on the roof of the rooms and hallways so that employees, patients and visitors would not notice this hardware infrastructure. This is an important advantage over other RFID technologies that require the users to come closer to RFID readers (e.g., NFC, HF RFID).

Physical layer characterization, and, in particular, assessment of the radioelectric coverage should be taken into account when deploying UHF RFID systems, as reflections in walls and objects (especially in those with large metallic surfaces) may result in unexpected readings of tags outside the designed coverage area. Depending on the scenario, a basic coverage analysis as the one described in Section 2.1 can be enough. RSS information of the backscattered signal could be used to ensure that the detected RFID tag is actually within the coverage area of the RFID antenna. For example, RSS values could be stored in the database of the system and, if they are below a certain threshold, a message could be displayed (e.g., “item #n not detected properly. Please place it in another location”).

As mentioned in Section 1, one of the design goals of IoT-based systems for management and logistics is to minimize human interaction with the system, not only increasing the level of comfort of the users, but also minimizing the risk of mistakes. Even though this kind of RFID system is conceived to increase the level of automation of routine tasks such as drug administration, it still requires a minimum interaction from the users (e.g., pressing the button “medicine successfully administered” in the case of the example presented in this contribution), and, as stated in Section 1, this procedure still has some risk of human failure/mistake (e.g., pressing the button in advance, and then not administering the drug). Nevertheless, these RFID systems can also contribute to making the location and identification of medical errors easier, as they provide location and time information about the items registered within the system (medical assets, medicines, etc.).

Another possible improvement, already introduced in other areas where RFID systems are used for management and logistics could be related to automatic registration and removal of medical assets in the database, without requiring an end-user to manually insert the information associated to each item. These procedures can be implemented by placing several RFID antennas and readers devoted to registering new medical items, so every time a RFID tag is read by these antennas a new register in the database is created. Similarly, several RFID antennas and tags can be located in the area devoted to medical assets and drug disposal: every time an RFID tag is read in this area of the hospital, the item is deleted from the database.

## 5. Conclusions

This contribution has revised the impact of RFID systems for reducing the risk of medical errors in hospitals, especially in the field of drug administration. The added value comes from the hardware point-of-view, discussing advantages and limitations of different kinds of RFID technology, and analyzing the coverage area and the effects of multipath in the case of UHF RFID systems. A proof-of-concept based on UHF RFID hardware is presented, proposing a basic architecture for tracking medical items and drugs, which can be easily integrated within medical information services and network infrastructure already deployed in the majority of hospitals. A mobile application has been implemented to show real-time information about the items tagged with RFID tags, aiming to make it as simple as possible so every potential user can understand how it works almost immediately.

Tests conducted in a hospital have shown that UHF RFID technology can be successfully used to detect passive RFID tags at distances up to 3–4 m even in NLOS conditions, requiring similar hardware needs as HF RFID or NFC-based management systems. Thanks to its longer reading distance, UHF RFID has less impact than other RFID technologies, not requiring the users of the system (doctors, patients, nurses) to approach the readers.

## Figures and Tables

**Table 1:** Summary of advantages and disadvantages of optical and radiofrequency technologies for people and asset tracking and monitoring [8,12,13,14,15,16].

	Optical	Radiofrequency	
	QR/barcodes	High-frequency (HF) radio frequency identification/near-field communication (RFID/NFC) (13.56 MHz)	Ultra high-frequency (UHF) RFID (868 MHz, 915 MHz)
Advan-tages	Reading technology based on image processing.	Can work even with non line-of-sight (NLOS) conditions between the reader and the tags.	
	Low cost of the readers.		High throughput (up to hundreds of tags read per second).
	Widespread and mature technology, users worldwide are familiarized with it.		
			Reading range up to 10 m.
			Capability to read several tags simultaneously.
Disadvan-tages	Can require a person to operate (increasing the amount of work, or the risk of mistakes).		Moderate to high cost of UHF RFID readers and antennas.
	Limited reading range. Requires line-of-sight (LOS) between reader and the QR/barcode.	Limited reading range (25–50 cm).	
			Can be affected by multipath, interferences (reducing RFID system throughput and performance).
	Only one barcode/QR code can be read at a time.		
	Read-only tags.		
	Subject to environmental conditions (good visibility and illumination).		

**Table 2:** Review of RFID-based systems for management and tracking in hospitals.

RFID System/Hospital	RFID Technology	Connectivity	Application
Bon Secours hospitals. Richmond, USA [17].	HF RFID (13.56 MHz)	Wireless local area network (WLAN)/hospital network	Location and tracking of medical assets and patients.
Multi-purpose hub application. A Coruña hospital, Spain [18].	Hybrid system: NFC (13.56 MHz), UHF RFID (868 MHz).	WLAN	Management and tracking of patients and medication.
MASCAL. San Diego, USA [19].	HF RFID (13.56 MHz)	WLAN	Management of resources at a hospital during a mass casualty situation.
RFID-based real-time location system (RTLS) [20]. Benjamin Rusell Hospital. Alabama, USA.	Hybrid infrared/RFID system	WLAN/hospital network	Location of medical items requiring maintenance. Nurse-call system
Liverpool Hospital. Liverpool, Australia [21].	HF RFID (13.56 MHz)	WLAN/hospital network	Blood supplies tracking and management.

## Supplementary Materials

The following are available online at http://www.mdpi.com/1424-8220/18/8/2663/s1.
